# Evidence for substrate-assisted catalysis in *N*-acetylphosphoglucosamine mutase

**DOI:** 10.1042/BCJ20180172

**Published:** 2018-08-16

**Authors:** Olawale G. Raimi, Ramon Hurtado-Guerrero, Daan M.F. vanAalten

**Affiliations:** Division of Gene Regulation and Expression, School of Life Sciences, University of Dundee, Dundee DD1 5EH, U.K.

**Keywords:** *Aspergillus fumigatus*, catalysis, phosphohexomutase, reaction mechanism

## Abstract

*N*-acetylphosphoglucosamine mutase (AGM1) is a key component of the hexosamine biosynthetic pathway that produces UDP-GlcNAc, an essential precursor for a wide range of glycans in eukaryotes. AGM belongs to the α-d-phosphohexomutase metalloenzyme superfamily and catalyzes the interconversion of *N*-acetylglucosamine-6-phosphate (GlcNAc-6P) to *N*-acetylglucosamine-1-phosphate (GlcNAc-1P) through *N*-acetylglucosamine-1,6-bisphosphate (GlcNAc-1,6-bisP) as the catalytic intermediate. Although there is an understanding of the phosphoserine-dependent catalytic mechanism at enzymatic and structural level, the identity of the requisite catalytic base in AGM1/phosphoglucomutases is as yet unknown. Here, we present crystal structures of a Michaelis complex of AGM1 with GlcNAc-6P and Mg^2+^, and a complex of the inactive Ser69Ala mutant together with glucose-1,6-bisphosphate (Glc-1,6-bisP) that represents key snapshots along the reaction co-ordinate. Together with mutagenesis, these structures reveal that the phosphate group of the hexose-1,6-bisP intermediate may act as the catalytic base.

## Introduction

*N*-acetylphosphoglucosamine mutase (AGM1) catalyzes the interconversion of *N*-acetylglucosamine-6-phosphate (GlcNAc-6P) to *N*-acetylglucosamine-1-phosphate (GlcNAc-1P) in eukaryotes [1]. This enzyme is a member of the α-d-phosphohexomutase superfamily of enzymes that catalyze intramolecular phosphoryl transfer on a range of phosphosugar substrates [2]. The reaction catalyzed by AGM1 is the third step in the biosynthesis of UDP-GlcNAc from fructose-6-phosphate in the hexosamine pathway [3]. UDP-GlcNAc is an important metabolite for many cellular processes. In bacteria, it is the precursor of outer membrane lipopolysaccharide [4,5] and cell wall peptidoglycan [6]. In eukaryotes, it is the precursor of GPI anchors [7] together with its role as sugar donor in many glycosylation reactions including N-glycosylation and O-glycosylation [8,9]. In fungi, UDP-GlcNAc is also the precursor of chitin and mannoproteins, which are essential components of the fungal cell wall [3]. Knockout of any of the genes encoding enzymes of the UDP-GlcNAc pathway has been demonstrated to be lethal in *Saccharomyces cerevisiae*, unless exogenous glucosamine or *N*-acetylglucosamine is available [1,10,11]. Previously, AGM1 was shown to be an essential gene in the human fungal pathogen *Aspergillus fumigatus* and has been proposed as a drug target [12]. However, there are no mechanism-inspired inhibitors of this family of enzymes due to a limited understanding of their catalytic mechanisms.

The phosphohexomutase superfamily comprises two subfamilies separated by their ability to operate on α-d-sugar 1-phosphates or β-d-sugar 1-phosphates. While the former family is distinguished by the phosphorylation of a conserved serine to give a stable phosphate monoester [2], the latter uses a conserved aspartate as a nucleophile to form a transient phospho-enzyme intermediate [13]. Some members of the phosphohexomutase superfamily, phosphoglucomutases, bacterial phosphoglucosamine mutases and yeast phosphoacetylglucosamine mutases share the common sequence motif Ser/Thr-X-Ser-His-Asn-Pro [14,15]. Phosphorylation of these enzymes, shown to occur on the serine at the third position in the sequence motif [16], is required for full activity; however, the enzymes are initially produced in an inactive, dephosphorylated form [17]. A hexose-1,6-bisphosphate has been reported to be the phosphate donor in the activation process during catalysis that occurs via a ping-pong bi-bi mechanism [14,16,18]. It has also been shown that the phosphoglucosamine mutase from *Escherichia coli* autophosphorylates *in vitro* in the presence of [^32^P]ATP and the same was observed for phosphoglucosamine mutases from other bacterial species, yeast *N*-acetylglucosamine-phosphate mutase, and rabbit muscle phosphoglucomutase [17]. Several crystal structures complexed with phosphate mimics (e.g. trifluoromagnesate ‘MgF_3_^−^’ and trifluoroberrylate ‘BeF_3_^−^’) or phosphate together with phosphosugars have revealed the residues implicated in substrate binding and catalysis, and revealed large conformational changes [2,13,19]. However, the identity of the catalytic base for these enzymes is as yet unknown.

Here, we describe the crystallographic snapshots of *A. fumigatus* AGM1 (*Af*AGM1) that reveal large conformational changes accompanying phosphoryl transfer. Furthermore, the complexes, together with modelling studies, suggest that the phosphate group of the reaction intermediate may act as the catalytic base during catalysis. This increase in understanding of the reaction mechanism of this class of enzymes could be exploited in the rational design of mechanism-inspired inhibitors.

## Materials and methods

### Mutagenesis

The previously published *Af*AGM1 GST fusion construct, pGEX-6P *Af*AGM1 [12], served as the template for the generation of S69A and S507A amino acid substitutions by site-directed mutagenesis, using the mutagenic oligonucleotides 5′-GGATTGGCGTCATGGTCACTGCGGCTCATAATCCTGCCGAGGACAATGG-3′ (S69A forward), 5′-CCATTGTCCTCGGCAGGATTATGAGCCGCAGTGACCATGACGCCAATCC-3′ (S69A reverse), 5′-GGGACGAAGCTTCGCTCGTGCAG7CTGGCACGGAAGATGCGGTGCGTG-3′ (S507A forward) and 5′-CACGCACCGCATCTTCCGTGCCAGCTGCACGAGCGAAGCTTCGTCCC-3′ (S507A reverse). Site-directed mutagenesis was carried out following the QuikChange Site-Directed Mutagenesis protocol (Stratagene), using the KOD HotStart DNA polymerase (Novagene). All plasmids were verified by sequencing using the University of Dundee sequencing service.

### Protein production and purification

*Af*AGM1 and mutated forms were produced and purified as described previously [12]. Briefly, the *E. coli* strain BL21 (DE3) pLysS was used to produce the proteins and the purification consisted of chromatographic steps, with glutathione Sepharose 4B beads and a Superdex75 gel filtration column (2.6 × 60 cm) (Amersham Biosciences). Prior to gel filtration, the GST from the fusion protein was cleaved by PreScission protease.

### Crystallization, data collection, and structure determination

Crystals of the wild-type enzyme were grown in a mother liquor containing 20% PEG 1000 and 100 mM HEPES (pH 7.25), as described previously [12]. The complex with GlcNAc-6P was obtained by soaking experiments with the sugar and an excess of MgCl_2_. Crystals of the *Af*AGM1_S69A mutant co-crystallised with 15 mM Glc-1,6-bisP and 5 mM MgCl_2_ were obtained in several conditions: 0.2 M sodium thiocyanate, 20% PEG 3350, and 0.1 M sodium bromide; 0.10 M glycine, 22% PEG 3350, and 0.2 M sodium thiocyanate; and a final condition with 0.16 M glycine, 16% PEG 3350, and 0.2 M sodium thiocyanate. The two complexes crystallised after 2–3 days in the space group *P*2_1_2_1_2_1_ with differences in the unit cell dimensions with respect to the wild type (Table 1). X-ray diffraction data were collected at the BM14 beam line of the European Synchrotron Radiation Facility (ESRF, Grenoble, France). Single crystals were cryoprotected (15% glycerol plus the appropriate mother liquor) and frozen in a nitrogen gas cooled to 100 K. Complete data sets were collected for the GlcNAc-6P and Glc-1,6-bisP complexes. Data were processed with HKL2000 [20]. Both complexes were solved by molecular replacement using a previously described structure of the wild-type enzyme in complex with magnesium [12]. Refinement was performed with REFMAC5 [21] and model building with COOT [22]. Ligand structures and topologies were generated by PRODRG [23]. Models for ligands were not included until their conformations were completely defined by unbiased |*F*_o_| − |*F*_c_|, *φ*_calc_ electron density maps. Pictures were generated using Pymol [24].

**Table 1:** Details of data collection and structure refinement Values between the brackets are for the highest resolution shell. All measured data were included in structure refinement.

	*Af*AGM1–GlcNAc-6P	*Af*AGM1_S69A–Glc-1,6-bisP
Resolution	20.00 (2.34)	20.00 (1.90)
Space group	*P*2_1_2_1_2_1_	*P*2_1_2_1_2_1_
Unit cell		
*a* (Å)	71.3	80.9
*b* (Å)	84.8	87.6
*c* (Å)	186.4	91.9
No. of reflections	183 592	191 366
No. of unique reflections	48 393	51 704
*I*/*σ* (*I*)	34.7 (1.8)	19.2 (2.7)
Completeness (%)	99.8	97.6
Redundancy	3.5 (3.9)	3.7 (3.6)
*R*_merge_ (%)	5.7 (66.6)	3.4 (48.2)
RMSD from ideal geometry		
Bonds (Å)	0.010	0.012
Angles (°)	1.5	2.1
*R*_work_ (%)	21.0	16.0
*R*_free_ (%)	27.7	20.0
No. of molecules in a.s.u	2	1
No. of residues	1036	541
No. of water molecules	297	471
*B* factors (Å^2^)		
Overall	52.4	26.9
Protein	52.6	26.3
Ligand	56.4	30.6
Solvent	48.2	31.9
PDB ID	5OAW	5O9X

### Enzyme kinetics

**Figure 1.**
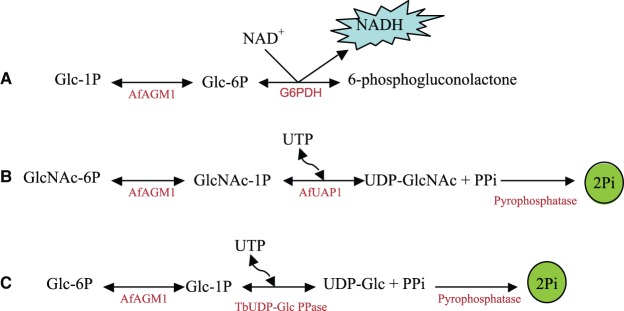
Schematic illustrations of *Af*AGM1 assays. (**A**) Phosphoglucomutase assay: a coupled assay with glucose-6-phosphate dehydrogenase (G6PDH) using G-1P as a substrate. (**B**) Phosphoglucosamine mutase assay: a coupled assay with *A. fumigatus* UDP-*N*-acetylglucosamine pyrophosphorylase (*Af*UAP1) and pyrophosphatase using GlcNAc-6P as a substrate. (**C**) A reverse phosphoglucomutase assay: a coupled assay with *Trypanosoma brucei* UDP-glucose pyrophosphorylase (*Tb*UGP) and pyrophosphatase using G-6P as a substrate.

To determine the phosphoglucose mutase and phophoglucosamine mutase activities of AGM1, three different coupled assays were carried out (Figure 1). Assay A was carried out in the presence of glucose-6P dehydrogenase (G6PDH) with or without Glc-1,6-bisP as a cofactor using glucose-1P (Glc-1P) as the substrate. This assay was carried out in a 100 µl reaction volume containing 50 mM MOPS (pH 7.4), 1.5 mM MgSO_4_, 1 mM DTT (dithiothreitol), a range of Glc-1P concentrations, 1 mM NAD^+^, and 0.01 units of G6PDH (Sigma). The reaction was started by the addition of 10 nM *Af*AGM1 and incubated for 60 min at 20°C. The amount of NADH produced was measured using a fluorescence reader (FL×800). Assay B was done in the presence of UDP-*N*-acetylglucosamine pyrophosphorylase (UAP1) as the coupled enzyme using GlcNAc-6P as the substrate as described previously [12]. The reaction mixture (100 µl) contained 50 mM MOPS (pH 7.4), 1.5 mM MgSO_4_, 250 µM UTP, varying concentrations of GlcNAc-6P (2.5–300 µM), 100 nM *Af*AGM1, 0.5 µM *Af*UAP1, and 0.04 units pyrophosphatase (Sigma) to convert the *Af*UAP1 reaction product inorganic pyrophosphate (PPi) to inorganic phosphate (Pi). The reaction was incubated at 20°C for 30 min and terminated by the addition of 100 µl Biomol green [0.03% (w/v) malachite green, 0.2% (w/v) ammonium molybdate, and 0.5% (v/v) Triton X-100 in 0.7 N HCl] and left for a further 20 min at 20°C for colour development. Absorbance at 620 nm was read using a spectrophotometer. Assay C involves coupling with UDP-glucose pyrophosphorylase using glucose-6P (Glc-6P) as the substrate and the Biomol green assay as described earlier [12,25].

### Modelling and energy minimization of AfAGM1_S69A mutant–Glc-1,6-bisP complex

To generate a model of the complex of *Af*AGM1 with Glc-1,6-bisP, the *Af*AGM1_S69A–Glc-1,6-bisP mutant complex crystal structure was used as a template. Using COOT, the Ala69 in *Af*AGM1_S69A mutant was mutated to the original Ser69 and a rotamer of Asp288 was chosen to allow coordination of the magnesium as found in the wild-type structure with Mg^2+^ and GlcNAc-6P (see below). Magnesium was also incorporated in COOT after superposition with the wild-type structure with Mg^2+^ and GlcNAc-6P complex, and energy minimization was performed using the Schrödinger software [26]. The crystal structure was read with the Maestro program [27], and the Protein Preparation Wizard module was used to add hydrogens, assign correct bond orders, and solve steric conflicts. Once the structure was prepared, a partial minimization of hydrogen atoms was initially performed by means of the Impact Refinement module, using the OPLS-2005 force field [27], and finished when RMSD (root mean square deviation) reached a maximum cut-off of 0.3 Å. After this initial minimization, a further total minimization including heavy atoms was also made using the same conditions and terminated with an RMSD of 0.10 Å with respect to the original *Af*AGM1_S69A mutant–Glc-1,6-bisP complex.

## Results and discussion

### Ser69 is the *Af*AGM1 catalytic serine

**Figure 2.**
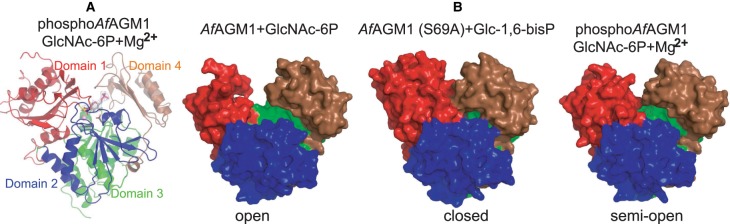
Overall structure of *Af*AGM1. (**A**) Overall crystal structure of one of the two monomers found in the asymmetric unit of *Af*AGM1–GlcNAc-6P–Mg^2+^ ternary complex. Domains 1, 2, 3, and 4 are coloured in red, blue, green, and brown, respectively. The carbon sticks of *Af*AGM1_pSer69 and GlcNAc-6P are coloured in yellow and grey, respectively. (**B**) Surface representation of the monomeric forms of *Af*AGM1 in complex with GlcNAc-6P and Glc-1,6-bisP that show different conformational states during catalysis (colours of the different domains are the same as above).

**Figure 3.**
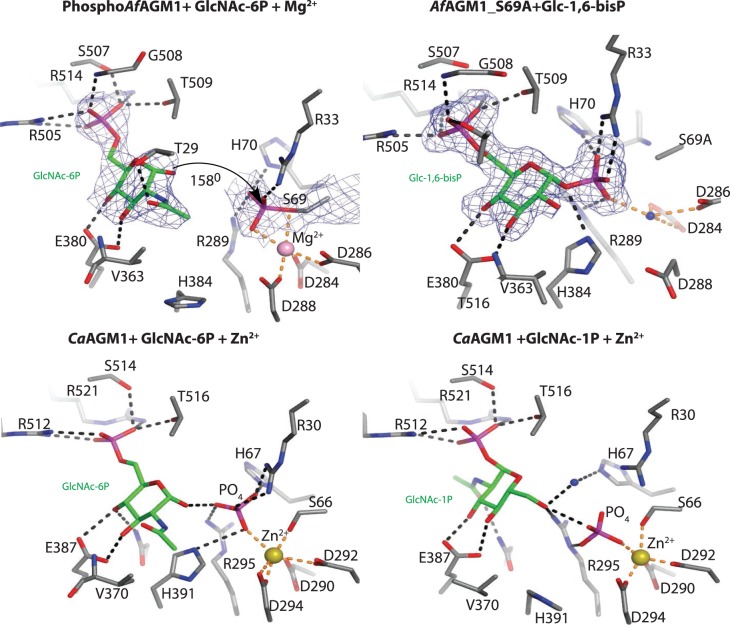
Active sites of *Af*AGM1 and *Ca*AGM1 in complex with different ligands. The active sites of *Af*AGM1–GlcNAc-6P–Mg^2+^ and *Af*AGM1_S69A–Glc-1,6-bisP complexes are compared with *Ca*AGM1–GlcNAc-6P–Zn^2+^-phosphate (PDB 2DKC) and *Ca*AGM1–GlcNAc-1P–Zn^2+^-phosphate (PDB 2DKD) complexes [2]. Carbon atoms of residues within the active site pocket and sugars are shown as grey and green sticks, respectively. Mg^2+^ and Zn^2+^ ions, and water molecules are shown as pink, yellow, and blue spheres, respectively. Hydrogen bond interactions between the protein and the ligands are shown as black dashed lines, while Mg^2+^ and Zn^2+^ co-ordinations are shown as brown dash lines. The |*F*_o_| − |*F*_c_|, *φ*_calc_ electron density maps around the ligands are shown contoured at 2.5 *σ* and also the final 2|*F*_o_| − |*F*_c_| electron density map for the phosphorylated active Ser69 is shown, contoured at 1.0 *σ*. A black arrow indicates the inline angle of attack of 158° of 1-hydroxyl of GlcNAc-6P on the phosphate located in *Af*AGM1_pSer69.

Previously, we identified pSer69 as the potential catalytic residue in *Af*AGM1 by mass spectrometry (MS/MS), phosphosite mapping, and crystallographic studies of the wild-type enzyme in complex with magnesium [12]. To further explore the role of the *Af*AGM1_pSer69 in catalysis, we determined steady-state kinetics with different substrates in the absence and presence of Glc-1,6-bisP as the activator. The wild-type enzyme demonstrated both *N*-acetylphosphoglucosamine and phosphoglucose mutase activity with *K*_m_ values of 25 µM for its native substrate GlcNAc-6P, 300 µM for Glc-6P, and 1.2 mM for Glc-1P (Table 2), demonstrating that the enzyme specifically recognizes the *N*-acetyl group. We also determined the crystal structure of *Af*AGM1 in complex with GlcNAc-6P and magnesium (Table 1), showing the typical four domains forming a heart shape found previously for other members (domains 1–4, Figure 2A,B). Domains 1 (residues 1–187), 2 (residues 188–305), 3 (residues 306–442), and 4 (residues 443–542) bear the predicted active serine loop, the metal-binding loop, the sugar-binding loop, and the phosphate-binding, respectively. The overall structure of this enzyme is similar to that of *Candida albicans N*-acetylphosphoglucosamine mutase (*Ca*AGM1) [2] (52% sequence identity, RMSD of 1.2 Å on 496 Cα atoms) and the *Pseudomonas aeruginosa* phosphomannomutase/phosphoglucomutase (*Pa*PMM/PGM) [28] (20% sequence identity and RMSD of 2.3 Å on 343 Cα atoms). At the active site of one of the two monomers in the asymmetric unit, unambiguous electron density defining a phosphorylated Ser (Ser69) was present (Figure 3A). The conformation of the phosphoserine with an O…..P–O angle of 158° would facilitate inline attack/displacement by the GlcNAc-6P O1 and hence further supports the role of this residue as the phosphoryl donor/acceptor (Figure 3A).

**Table 2:** Kinetic parameters of *Af*AGM1 The coupled assay with G6PDH (Figure 1) was used to measure *Af*AGM1 activity using Glc-1P as the substrate in the presence or absence of Glc-1,6-bisP. The colorimetric assay involving purified *Af*UAP1 or *Trypanosoma brucei* UDP-glucose pyrophosphorylase as coupling enzymes (Figure 1) was used to measure *Af*AGM1 activity using GlcNAc-6P or Glc-6P as the substrate, respectively. The results are the mean ± SD for three determinations. Abbreviations: n.d.: not detectable. The values for the wild-type enzyme have been reported earlier but are shown here for comparison purposes with *Af*AGM1_S69A and *Af*AGM1_S507A mutants [12].

Substrate	WT	S69A	S507A
Glc-1P			
*K*_m_ (μM)	1200 ± 100		1900 ± 100
*V*_max_ (μmol/s)	0.38 ± 0.01		0.138 ± 0.004
*k*_cat_ (s^−1^)	37.6	n.d.	13.8
Glc-1P + Glc-1,6P			
*K*_m_ (μM)	400 ± 30		1200 ± 100
*V*_max_ (μmol/s)	0.41 ± 0.01		0.121 ± 0.004
*k*_cat_ (s^−1^)	41.0	n.d.	12.1
GlcNAc-6P			
*K*_m_ (μM)	25 ± 8		25 ± 9
*V*_max_ (μmol/s)	0.021 ± 0.002	n.d.	0.008 ± 0.001
*k*_cat_ (s^−1^)	0.21		0.08
Glc-6P			
*K*_m_ (μM)	300 ± 48		2900 ± 600
*V*_max_ (μmol/s)	0.018 ± 0.001		0.011 ± 0.001
*k*_cat_ (s^−1^)	0.18	n.d.	0.11

To further verify the role of S69 as the phosphoryl donor/acceptor, the S69A *Af*AGM1 mutant was investigated. As expected, steady-state kinetics (Table 2) reveal that mutating Ser69 to alanine completely abolishes enzyme activity. A similar study of human AGM1 also reported a complete loss of activity when the equivalent Ser64 was mutated to alanine or threonine [29]. Interestingly, in a study of an equivalent mutant (Ser108Ala) in *Pa*PMM/PGM, another member of the same phosphohexomutase superfamily [30,31], some residual activity was reported [31]. It was proposed that this might be the result of another residue in the active site being phosphorylated in the absence of Ser108 [30]. We noted the proximity of Ser507 to the substrate-binding site, located in the phosphate-binding loop (Figure 3). However, mutating the *Af*AGM1 S507 to alanine does not significantly affect activity (Table 2), suggesting that this serine does not contribute to catalysis. Thus, Ser69 is the *Af*AGM1 catalytic serine.

### Phosphoryl transfer is associated with large conformational changes

Once it was clarified that Ser69 was the only residue being phosphorylated during catalysis, we exploited this catalytically impaired mutant to trap the bisphosphorylated intermediate that has been proposed for this class of phosphomutase [30] (Figure 3). The protein was crystallized with Glc-1,6-bisP and the structure was refined to 1.9 Å resolution with a single protein molecule in the asymmetric unit (Table 1). The overall structure of *Af*AGM1_S69A–Glc-1,6-bisP complex shows a closed conformation, compared with the open/semi-open conformations exhibited by the two monomers in the *Af*AGM1–GlcNAc-6P–Mg^+2^ complex (Figure 2B). Together, these complexes reveal overall conformational changes accompanying phosphoryl transfer. Superposition of the opened and semi-opened conformations yields an RMSD of 0.76 Å with the loop on domain 4 moving 1.4 Å towards the active site cleft (Supplementary Figure S1). Superposition of the semi-opened and closed conformations yields an RMSD of 2.1 Å with the loops on domains 1 and 4 moving 2.0 and 3.4 Å, respectively. Superposition of the opened and closed conformations yields an RMSD of 2.3 Å with the loop on domain 4 moving 4.0 Å, while the loop on domain 1 is disordered in the opened conformation structure (Supplementary Figure S1). Thus, domain 4 of the enzyme appears to be mobile, closing, or opening the active site in response to interactions between the enzyme and its substrates/products/intermediate. We have visualized this with a video that has been included in Supplementary Figure S2. The dephospho-*Af*AGM1–GlcNAc-6P complex has an overall structure with an open active site (Figure 2B) and may represent the inactive enzyme or ‘apo’ like conformation. This complex provides the likely snapshot of the conformation of the protein just before the first phosphoryl transfer or the release of product from the active site. However, the inactive *Af*AGM1_S69A mutant–Glc-1,6-bisphosphate complex has an overall structure with a closed active site (Figure 2B) and represents the reaction intermediate with Glc-1,6-bisphosphate buried in the active pocket. This is the first crystallographic evidence of the structural changes by this class of enzymes during catalysis, which corroborates the reaction mechanism proposed by Regni et al. [28]. The complete closure of the active site is achieved by the establishment of extensive hydrogen bond interactions between the reaction intermediate Glc-1,6-bisphosphate and the protein (Figure 3). While the 1-phosphate group of the ligand pointing to Ala69 occupies partly the position of the phosphoserine observed in the *Af*AGM1_S69A–Glc-1,6-bisP complex, the 6-phosphate group is located in the phosphate-binding domain. In addition, the 1-phosphate group establishes hydrogen bond interactions with R33, H70, R289 and a water molecule, the 6-phosphate group makes hydrogen bond interactions with T29, R505, S507, T509, R514 and the backbone of G508, and the 3-OH and 4-OH groups of the sugar make hydrogen bond interactions with E380 and the backbone of V363 (Figure 3). Thus, extensive hydrogen bond interactions are established with residues from all the domains resulting in a closed conformation (Figure 2B). This provides a snapshot of the reaction either immediately after phosphoryl transfer to form the intermediate or before the second phosphoryl transfer to re-phosphorylate the enzyme. The phospho-*Af*AGM1–GlcNAc-6P–Mg^2+^ Michaelis complex reveals a semi-opened active site and may represent the active complex. For this complex, it was observed that GlcNAc-6P made hydrogen bond interactions with residues R505, R514, S507, T509, G508, E380 and V363 from domains 3 and 4 of the protein but not with any residues from domains 1 and 2. Furthermore, the *Af*AGM1_pSer69 establishes hydrogen bond interactions with residues R33, R289 and H70 from domains 1 and 2 as well as Mg^2+^, which is co-ordinated by D288, D284 and D286 (Figure 3); hence, domains 3 and 4 relax to open the active site cleft (Figure 2B). Although these interactions are different from the previously determined *Ca*AGM1–GlcNAc-6P–Zn^2+^–P or *Ca*AGM1–GlcNAc-1P–Zn^2+_^P complex [2] which shows extensive hydrogen bond interactions with residues from all the domains (Figure 3), the phospho-*Af*AGM1–GlcNAc-6P–Mg^2+^ complex may represent a snapshot of the conformation that supports the proposed reorientation of the intermediate or conformation just before the first phosphoryl transfer (Figure 2B). It is likely that for the reorientation of the intermediate to occur, the domains have to be relaxed from the closed state to the semi-open conformation (Figure 2B) allowing the reorientation of the intermediate without leaving the enzyme active site. In summary, these structures reveal that phosphoryl transfer is associated with large conformational changes.

### The catalytic role of Ser69 depends on its coordination to a magnesium ion

AGM belongs to the α-d-phosphohexomutase metalloenzyme superfamily and the magnesium ion is essential for catalysis [16]. Structures of related phosphohexomutases in complex with substrates/products or the bisphosphosugar intermediate have been described, but they have not provided a detailed understanding of the atomic trajectories during catalysis [28,32]. In this work, we have obtained two different complexes that represent distinct steps of the catalytic cycle. At the active site level, both pSer69 and phosphate group either present in Glc-1,6-bisP or as a free phosphate in *Ca*AGM1 [2] interact by electrostatic interactions and hydrogen bonds with conserved residues such as Arg33, His70, Arg289, and H384 (Figure 3; in *Ca*AGM1 these residues correspond to Arg30, His67, Arg295, and His391, respectively). His384 (His391 in *Ca*AGM1) is the only residue adopting on and off conformations that supports the concept of ‘near attack conformers (NACs)’ present in other members of this superfamily [13]. These conformers exist to avoid repulsion among highly charged residues and consequently to optimize catalytic turnover [13]. In the case of AGM1, this histidine may switch from an off to an on conformation to activate the O-1 hydroxyl group of the GlcNAc-6P that would subsequently attack the phosphate atom (Figure 3). Furthermore, the phosphate group is co-ordinated either by a magnesium ion (see the phospho-*Af*AGM1–GlcNAc-6P–Mg^2+^ complex in Figure 3), which is needed in turn to have an active enzyme, or a zinc ion acting as an AGM1 inhibitor (see the *Ca*AGM1–ligand complexes in Figure 3) [2]. In *Af*AGM1, the magnesium ion is pentagonally co-ordinated in a square pyramidal arrangement by *Af*AGM1_pSer69, Asp284, Asp286, and Asp288 (Figure 3). This type of coordination has also been described for the structure of *Ca*AGM1 complexed with either GlcNAc-1P or GlcNAc-6P and zinc acting as an inhibitor (Figure 3) [2]. Although the inactive *Af*AGM1_S69A mutant–Glc-1,6-bisphosphate complex was crystallized in the presence of 10 mM MgCl_2_, the structure (Figure 3) reveals the absence of this metal and the presence of a water molecule instead (Figure 3). The magnesium ion helps to stabilize the charges and hence facilitates the transfer of phosphoryl group between the *Af*AGM1_pSer69 and the O1 or O6 of the substrates [2,33]. The catalytic serine is, however, key in the coordination to the magnesium ion throughout the catalytic cycle and therefore constitutes an essential feature for having a functional AGM1.

### The phosphate group as the putative catalytic base

**Figure 4.**
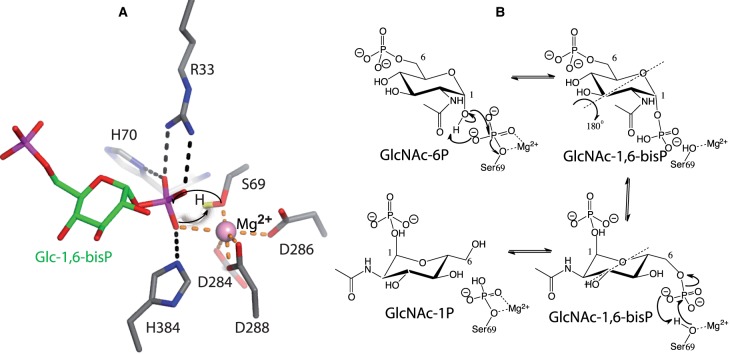
Catalytic mechanism. (**A**) Model of wild-type *Af*AGM1 active site in complex with Glc-1,6-bisP and magnesium. Colours are the same as Figure 3 and a hydrogen atom bound to Ser69 is shown in yellow. (**B**) Scheme of the proposed reaction mechanism of phosphoacetylglucosamine mutase.

Previous work on pSer-dependent phosphohexomutases combined with the two new structures shown in this study do identify the amino acids that may be required for activation of the O1/O6 hydroxyls groups. Inspection of some of the amino acids interacting with the phosphoserine, the Glc-1,6-bisP, or the free phosphate present in the *Ca*AGM1 [2] reveals many candidates that may fulfil this role. For instance, His70 or His384 and Arg33 that form hydrogen bond interactions with 1P group of Glc-1,6-bisP and phosphate group of *Af*AGM1_pSer69 (Figure 3) could act as activators. Another alternative is that both the hydroxyl group from Ser69 and the O1/O6 hydroxyls from GlcNAc-1P/6P act as a nucleophile in the absence of a catalytic base, although this is unlikely given the proximity of the positive charge on the magnesium ion. To further explore this, we made a model of the wild-type enzyme complexed with magnesium and Glc-1,6-bisP based on the structure of the inactive mutant reported here (Figure 4A). The template was used because it approximates the reaction intermediate catalytic step (Figure 3). Interestingly, this model suggests that the phosphates may uptake the proton from the hydroxyl group of Ser69 leading to an increase of nucleophilicity of the serine side chain that is required to attack the phosphate. One of the p*K*_a_s of this phosphate group is ∼6.8, which makes it compatible with its potential role as a catalytic base and further supports the proposed mechanism (Figure 4B). In this scenario, GlcNAc-6P enters the active site and the oxyanion of 1-hydroxyl from GlcNAc-6P is precisely formed once it is located in front of *Af*AGM1_pSer69 by proton uptake by the phosphate group. In this manner, negative repulsion of the substrate and *Af*AGM1_pSer69 would be avoided and the reaction will only take place when both are correctly positioned. GlcNAc-1,6-bisP (*N*-acetylglucosamine-1,6-bisphosphate) would be formed and the required flip over around the O5–C3 axis will render the 6-phosphate group in a position to be transferred to Ser69 by the same mechanism proposed (Figure 4B). Finally, GlcNAc-1P is formed, completing the catalytic cycle.

## Conclusions

*N*-acetylphosphoglucosamine mutase is a key enzyme of the hexosamine biosynthetic pathway in eukaryotes. The enzyme catalyzes the interconversion of GlcNAc-6P to GlcNAc-1P, which is subsequently converted to UDP-GlcNAc by UDP-GlcNAc pyrophosphorylase, the last enzyme in the pathway. UDP-GlcNAc is the direct precursor for many cellular processes, for instance, in fungi, it is used in the synthesis of chitin that forms the major component of the fungi cell wall and it is thus believed that enzymes involved in its synthesis are potential drug targets [3,34,35]. The results presented here have helped our understanding of the catalytic mechanism of *Af*AGM1 and the wider phosphohexomutase superfamily. *Af*AGM1 shares the same overall structure with other members of this superfamily although it is more similar to *Ca*AGM1 [2] (RMSD 1.2 Å) than to phosphomannomutase/phosphoglucomutase (RMSD 2.3 Å). Although the crystal structure of the well-characterized member of the phosphohexomutase superfamily *Pa*PMM/PGM with phosphorylated active Ser108 has been reported, here we describe the first crystal structure of a member of the phosphoacetylglucosamine mutase subfamily with phosphorylated active Ser69 (Figure 2A). This post-translational modification occurs during expression in *E. coli* by an as-yet-unknown mechanism which may involve autophosphorylation in the presence of ATP [17]. The kinetic characterization of the enzyme reveals that the enzyme possesses both phosphoglucomutase and phosphoacetylglucosamine mutase activity. The enzyme is capable of interconverting Glc-1P to Glc-6P, GlcNAc-6P to GlcNAc-1P, and Glc-6P back to Glc-1P but with different rate constants and GlcNAc-6P as the preferred substrate (Table 2). The structural complexes described in this study reveal that the enzyme adopts large conformational changes along the catalytic cycle. Thus, the active site of the dephospho-*Af*AGM1-GlcNAc-6P is open, representing an inactive state of the enzyme while the phospho-*Af*AGM1-GlcNAc-6P–Mg^2+^ adopts a semi-opened active site representing the active Michaelis complex. This complex, however, reveals that the mechanism of phosphoryl transfer from the active serine to the acceptor substrate might be due to the formation of an oxyanion of the 1-hydroxyl of GlcNAc-6P by proton uptake from *Af*AGM1_pSer69. Hence, a mechanism of phosphoryl transfer involving the ionization of the acceptor substrate by the phosphate acting as the catalytic base is the most plausible mechanism. In conclusion, these enzymes suffer large conformational changes, mainly in domain 4, which in turn are coupled to a precise phosphate-assisted mechanism in which phosphate may act as the catalytic base. The understanding of the catalytic mechanism can therefore be exploited in the rational design of a mechanism-inspired inhibitors which are lacking for this class of enzymes.

## Abbreviations

AfAGM1: *A. fumigatus* AGM1
AGM1: *N*-acetylphosphoglucosamine mutase
CaAGM1: *Candida albicans N*-acetylphosphoglucosamine mutase
G6PDH: glucose-6P dehydrogenase
Glc-1P: glucose-1P
Glc-6P: glucose-6P
GlcNAc-6P: *N*-acetylglucosamine-6-phosphate
PaPMM/PGM: *Pseudomonas aeruginosa* phosphomannomutase/phosphoglucomutase
RMSD: root mean square deviation
UAP1: UDP-*N*-acetylglucosamine pyrophosphorylase
