# Supplementary material for: Evidence for substrate-assisted catalysis in N-acetylphosphoglucosamine mutase
Source: Biochem J. 2018 Aug 16;475(15):2547–57. doi: 10.1042/BCJ20180172 (PMC6096347; doi:10.1042/BCJ20180172)

.

Supplementary Figure 1. **Superposition of the different *Af*AGM1 conformations**. This reveals movements of domains 1 and 4, with domain 4 making the greater movement towards the active site cleft. (A). *Af*AGM1_S69A+Glc1,6-bisP (Closed conformation) and phospho*Af*AGM1_GlcNAc-6P+Mg^{2+} (semi-open) with an RMSD of 2.1 Å. (B). *Af*AGM1+GlcNAc-6P (Open conformation) and phospho*Af*AGM1 GlcNAc-6P+Mg^{2+} (semi-open conformation) with an RMSD of 0.76 Å and (C). *Af*AGM1+GlcNAc-6P (open) and *Af*AGM1_S69A+Glc1,6-bisP (closed conformations), RMSD 2.3 Å. Coloured green is semi-opened, purple is closed and blue is opened conformations respectively. Figures 1 to 4 indicates the position of the domains and black arrows are pointing to the moving loops.

Supplementary Figure 2. Video showing the different stages of conformational change. Again, the secondary structures of domains 1, 2, 3 and 4 are coloured in red, blue, green and brown, respectively and the domain 4 showing the greatest movement.

Supplementary Figure 1

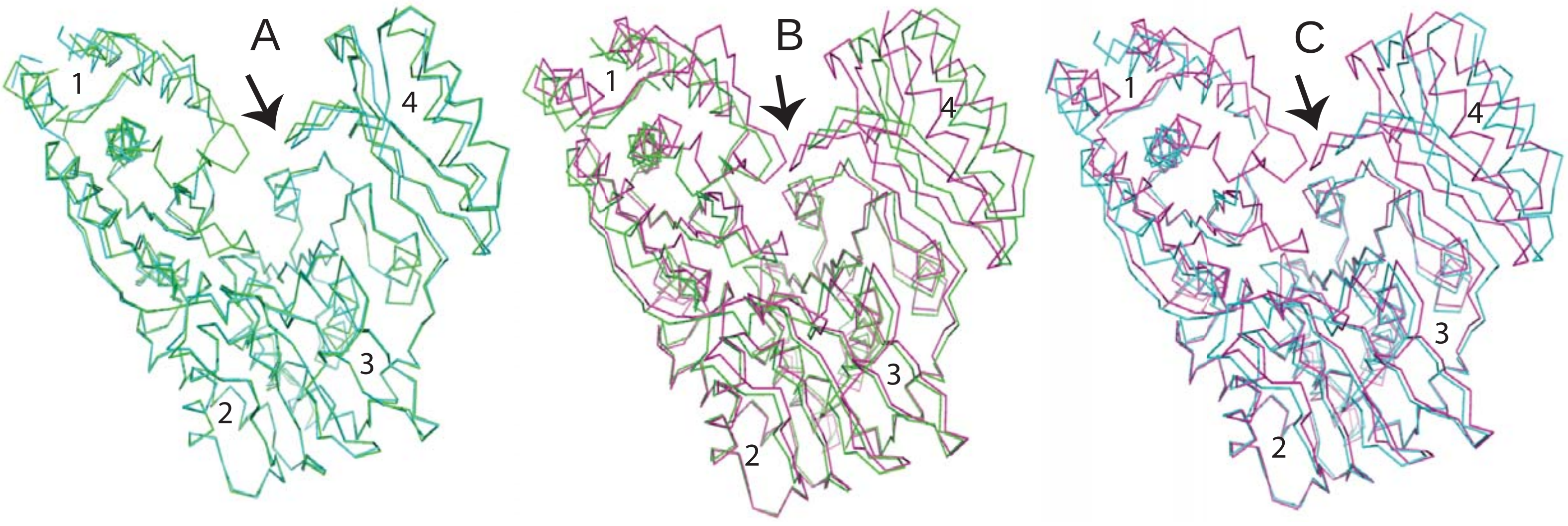

Supplement: Supplementary Figure S1 [file BCJ-475-2547-s1.pdf]
